# Healing effects of natural latex serum 1% from *Hevea brasiliensis* in an experimental skin abrasion wound model^☆^^☆☆^

**DOI:** 10.1016/j.abd.2019.12.003

**Published:** 2020-05-05

**Authors:** Marcel Nani Leite, Saulo Nani Leite, Guilherme Ferreira Caetano, Thiago Antônio Moretti de Andrade, Márcio Fronza, Marco Andrey Cipriani Frade

**Affiliations:** a Department of Clinical Medicine, Faculdade de Medicina de Ribeirão Preto, Universidade de São Paulo, Ribeirão Preto, SP, Brazil; b Department of Physiotherapy, Fundação Educacional Guaxupé, Guaxupé, MG, Brazil; c Graduate Program in Biomedical Sciences, Centro Universitário da Fundação Hermínio Ometto, Araras, SP, Brazil; d Universidade de Vila Velha, Department of Pharmacy, Graduate Program in Pharmaceutical Sciences, Universidade de Vila Velha, Vila Velha, ES, Brazil

**Keywords:** Dermabrasion, Inflammation, Latex, Wound healing

## Abstract

**Background:**

Dermabrasion is related with mechanical and surgical traumas on the skin; usually topical antiseptics and/or saline have been used for healing. Natural products for wound healing can also be used for abrasions, such as latex from *Hevea brasiliensis*.

**Objective:**

This study aimed to evaluate the *in vitro* viability and migratory/proliferative effects of latex serum from *H. brasiliensis* and to compare with a commercially available standard antiseptic solution and saline in experimental dermabrasion on rats.

**Methods:**

For *in vitro* evaluation, MTT and scratch assays were used. *In vivo* testing was performed in 72 rats submitted to dermabrasion, treated with saline, antiseptic, or latex serum. This study evaluated re-epithelialization, neutrophilic infiltration, and the quantification of crust and epidermis.

**Results:**

Latex showed viability at 1% and 0.1% concentrations and migratory/proliferative activity at 0.01% concentrations. The re-epithelialization was highest in latex group on 7th day. The latex group displayed lower thickness of crusts and greater extent of epidermal layers. The latex and antiseptic groups showed increases of myeloperoxidase levels on the 2nd day and showed important reductions from the 7th day.

**Study limitations:**

Acute superficial wound model in rats and non-use of gel-cream (medium) without latex.

**Conclusion:**

In conclusion, non-toxic latex stimulated migration/proliferation of keratinocytes *in vitro* and significantly accelerated wound healing in animal excoriation models compared to chlorhexidine or saline.

## Introduction

Cutaneous wounds are defined as the disruption of cellular and anatomical continuity of the skin and its functionality. They may involve the epidermis and/or dermis layers, and even muscles and bones.^1^, ^2^, ^3^ Acute tissue losses can arise after dermatological procedures, such as dermabrasion and chemical peels, or can be caused by physical trauma, essentially reaching the epidermis and surface of the dermis. In this case, tissue repair occurs only through re-epithelialization, followed by the process of anatomical and functional repair of the skin, resulting in an almost imperceptible scar. The healing time typically varies between five and ten days, but can also reach up to 30 days.^4^, ^5^ Usually, the injured skin areas are treated with topical products such as antiseptic solutions to protect and clean the skin when caused by domestic accidents, or just saline to maintain skin humidity after dermatological procedures avoiding the crust.

Medicinal products are used to restore health, in particular to accelerate the healing of cutaneous wounds. Extracts and oils from plants have demonstrated healing, antimicrobial, anti-inflammatory, and antioxidant effects. The literature shows several plants, such as *Calendula officinalis*, *Copaifera langsdorffii*, *Curcuma longa*, and *Chamaemelum nobile* (L.), among others.^6^, ^7^, ^8^, ^9^ However, medicinal plants are often used without scientific evidence, so further studies are needed to prove their efficacy and safety.^10^, ^11^, ^12^, ^13^

Among the substances from medicinal plants that have been investigated for their properties and contributions to healing processes, natural latex from the *Hevea brasiliensis* rubber tree is notable. Frade^14^ reported clinically evident signs of granulation stimulation and marked reduction in pain; these were confirmed by histopathological studies after 15 days of treatment with natural latex biomembrane (NLB). NLB has been found to act as an economic, easily handled, and highly effective dressing, mainly because of its debriding and angiogenic potential, permitting a dynamic and faster healing process, essential for wound healing.^15^

Several studies have demonstrated the role of latex protein with activity in wound healing both in nature and in serum, when compared with control groups.^15^, ^16^ Thus, considering the previous results using NLB for wound healing and the clinical importance related to the development of alternative and effective treatments for trauma and/or surgical excoriation, it was sought to compare the efficacy of usual treatments such as saline and antiseptic solution with latex serum (SLX) from the *H. brasiliensis* rubber tree in an experimental model of cutaneous excoriation in rats, since there are no other studies in the literature that show the healing effect in this model of excoriation.

## Methods

### Preparation of latex formulation and controls

Sterile sodium chloride solution 0.9% in water (S) and the antiseptic solution (AS) of chlorhexidine digluconate 10 mg/mL were purchased from a local pharmacy (Sao Paulo Pharmacy – Ribeirao Preto, Brazil). *H. brasiliensis* SLX was obtained from Pelenova Biotecnologia S/A Company (Distrito Industrial – Ribeirao Preto, Brazil) as described by Mendonça.^16^ Briefly, the latex was obtained from a rubber tree (*H. brasiliensis*) of the clone RRhim 600. Natural latex was treated with ammonium hydroxide (2%) when collected so that coagulation was avoided. A 2.2% acetic acid solution (1:2 v/v) was added under gentle stirring. In a next step, the pure serum was separated from the rubber and subjected to dialysis and lyophilization. The purification and characterization of the serum were performed as previously described to Mendonça^17^ using two-dimensional electrophoresis and mass spectrometry. Three main fractions were obtained, and named FrHB1, FrHB2, and FrHB3. Further, according the manufacturer of final product (gel-cream) used in pre-clinical experiments and also in clinical use, to eliminate the protein causing the greatest number of allergic reactions, hevein and its derivatives, the process of tangential filtration was performed to consider only substances below 10 kDa. Pure SLX was diluted in Dulbecco's Modified Eagle Medium (DMEM) at concentrations of 0.1–1% for the viability assay and 0.00001–1% for the migration/proliferation assay. For *in vivo* experiments, SLX in gel-cream was used (Valeant Pharmaceuticals International Inc. – São Paulo, SP, Brazil).

### Cell lines

NIH-3T3 (mouse fibroblast cell line) cells were purchased from the American Type Culture Collection (ATCC CRL-1658; Manassas – Virginia, United States). Human keratinocytes were isolated from healthy fragments of human skin with the informed consent of patients undergoing either breast or abdomen plastic surgery at the Ribeirão Preto Medical School, University of São Paulo, Ribeirão Preto, São Paulo, Brazil. All protocols were approved by the Research Ethics Committee of Ribeirão Preto Medical School, University of São Paulo, Ribeirão Preto, São Paulo, Brazil – process 5606/2008.

Fibroblasts were cultured in high-glucose DMEM (4.5 g/L; GIBCO – Invitrogen Corporation; Grand Island, NY, United States) and keratinocytes in Defined Keratinocyte Medium (GIBCO – Invitrogen Corporation; Grand Island, NY, United States) and were both supplemented with 10% fetal bovine serum and 1% antibiotic and antimycotic (GIBCO – Invitrogen Corporation; Grand Island, NY, United States), at 37 °C, in a humidified atmosphere containing 5% CO_2_.

### *In vitro* viability assessment

The viability of NIH-3T3 fibroblasts and human keratinocytes was determined by tetrazolium 3-[4,5-dimethylthiazol-2-yl]-2,5-diphenyl-bromide MTT colorimetric assay.^18^ Fibroblasts and keratinocytes were seeded in 96-well microplates at 2 × 10^4^ cells/mL concentration. Plates were incubated overnight for cellular adhesion. After this time, the supernatant was aspirated and 200 μL of test solutions containing SLX were added according to the following concentrations: only basal cell culture medium (positive control), 0.1% and 1% (SLX concentration was diluted in culture medium) and 50%/50% of culture medium and dimethyl sulfoxide (DMSO as negative control). Plates were incubated for 24 h and 48 h. After incubation, the supernatant was aspirated and the cells were washed with phosphate-buffered saline (PBS). Then, 20 μL of the stock solution of MTT (Sigma-Aldrich Co. LLC; 5 mg/mL in PBS) in 180 μL DMEM culture medium (without phenol red) was added to each well, and the plates were incubated under the same conditions for an additional 3 h. After that, the MTT solution was removed, and 200 μL of DMSO was added to dissolve the formazan crystals. Absorbance was read at 570 nm. The experiment was performed in triplicate, considering three independent experiments. The results were expressed as a percentage of cell viability.^18^, ^19^, ^20^

### *In vitro* cell migration/proliferation assay

To analyze the proprieties of SLX that stimulate keratinocyte migration/proliferation, an *in vitro* scratch assay was used.^21^, ^22^ Human keratinocytes were seeded in 24-well culture plates at a concentration of 3 × 10^4^ cells/well and were cultured with medium containing 10% fetal bovine serum until reaching a confluence of approximately 80%. Then, a linear scratch, mimicking an artificial wound, was created in the cellular monolayer using a 200 μL plastic tip. The medium was removed, and the cells were washed with PBS. Medium with 50% DMSO (negative control), culture medium with 10% fetal bovine serum (basal), set to 0 on the *X*-axis, and mediumS containing 1%, 0.1%, 0.01%, 0.001%, 0.0001%, or 0.00001% SLX concentrations were added in three wells per group and were incubated for 24 h at 5% CO_2_ at 37 °C. After 24 h, the wells were washed with PBS and were fixed with 4% paraformaldehyde for 15 min. The cells were washed again and stained with 4′,6-diamino-2-phenyl-indole (DAPI) for 5 min. Images of scraped areas were acquired using a fluorescence microscope coupled with a camera (Carl Zeiss® – Oberkochen, GE). The proliferation/migration of cells into the scraped region was quantified using ImageJ software (National Institutes of Health, Bethesda, MD, United States). The results were expressed as a percentage of cells that proliferate and/or migrate into the scratched area after SLX treatment compared to the number of migration/proliferation cells in control group. The experiments were also carried out in triplicate, considering also three independent experiments.

### Animals

Studies were conducted and approved by the ethical principles in animal research adopted by the Brazilian College of Animal Experimentation (COBEA) and approved by the Ethical Commission of Ethics in Animal Research (CETEA) – Ribeirão Preto Medical School, University of São Paulo, registry No. 072/2012.

A total of 72 adult male Wistar rats (*Rattus norvegicus*, 180–200 g) from the Central Animal Facility of Ribeirão Preto Medical School, São Paulo, Brazil, were housed for seven days before experimental process. During the entire experimental period, the rats were maintained in individual polycarbonate cages at a constant temperature (23 ± 2 °C) and humidity (55%) under a 12/12 h light/dark cycle. The animals had free access to a standard chow diet and drinking water.

### Surgical procedure and groups

Rats were anesthetized intraperitoneally with ketamine (70 mg/kg) and xylazine (12 mg/kg). The dorsum cervical region of each rat was trichotomized and cleaned with 70% alcohol. A dermoabrasor LB-100 device (BELTEC Indústria e Comércio de Equipamentos Odontológicos – Araraquara, SP, Brazil) was used to perform the excoriations with a diamond sanding disk RH14633 (RHOSSE Instrumentos e Equipamentos Cirúrgicos – Ribeirão Preto, SP, Brazil) on the back of the animal with a cutaneous lesion of approximately 2 cm^2^. Intraperitoneal dipyrone, at 50 mg/kg of body weight, was administered diluted in saline two times a day (12/12 h) during the first 48 h for preventing pain. Then, the animals were allocated in individual cages until the end of the experiment. The animals were divided into three groups, with 24 rats each, and treated daily with SLX 1%, a standard antiseptic solution (AS) (chlorhexidine digluconate 10 mg/mL) as a positive control, and saline (S) for ten days, without occlusive dressing.

### Evaluation of the re-epithelialization of abrasions

Images of the abrasion wounds were obtained on days 0, 2, 7, and 10 after treatment using a 14-megapixel digital camera (Kodak EasyShare M575) that was fixed in a standardized support for imaging, presenting a ruler as measurement reference and the calculation of the abrasion healing rate (AHR) was made by the following formula [(initial area − final area)/initial area] as described and performed by Leite^23^ for cutaneous wounds.

### Harvesting material

Eight animals from each group were euthanized using an excessive dose of anesthetic on days 2, 7, and 10. On each day of follow-up, a fragment was harvested with scissors. Half of the sample was embedded in 10% buffered formaldehyde solution (v/v) for histological study (hematoxylin–eosin); the other part of the sample wound was conditioned at −80 °C for biochemical assays.

### Measurement of the crust and epidermis

Biopsies were embedded in paraffin as previously described Andrade.^24^ A Leica DM 4000B optical microscope equipped with a LEICA DFC 280 camera (Leica Microsystems® – Germany) was used to capture the histological images at 400× magnification, using Leica Application Suite (LAS) software v. 3.2.0. One complete cut section of the lesion areas per animal (transversal section) was performed, in which three images were taken; three measurements were performed in each image. Both the measurements of the crust and epidermis were performed from one end to the other, mainly based on the neutrophil inflammatory infiltrate into the edge of epidermis. The image was opened and a line was drawn from the granular layer of the epidermis until the transition with the dermis (thickness of the epidermis) and thickness of the crust. At the end of the tracing, the software provided the distance in μm.^25^

### Measurement of myeloperoxidase (MPO)

The harvested biopsies were conditioned in 2 mL plastic tubes with 200 μL 0.1 M NaCl buffer, 0.02 M NaPO_4_, 0.015 M NaEDTA pH 4.7, and remained at −70 °C until use. The fragments were homogenized by an Omni Tissue Homogenizer (Kennesaw, GA, United States) at 13,000 rpm. After centrifugation, the fragments were resuspended in NaPO_4_ buffer (pH 5.4) containing 0.5% hexadecyltrimethylammonium bromide (HTAB). Then, 5 μL of the supernatant from the samples were placed in a 96-well plate for the assay. A standard curve for the neutrophils was obtained with isolated peritoneal mice neutrophils after 6 h of 0.25 mL carrageenan 1% (Sigma Aldrich) inoculation into the peritoneal cavity of the mice. Twenty-five microliters of 3,3′,5,5′-tetramethylbenzidine (TMB) (Sigma Chemical Company – St. Louis, MO, United States) was added to each well of the plate (samples and standard curve of the neutrophils), followed by 100 μL of H_2_O_2_. The reaction was then stopped with 4 M sulfuric acid and read on a plate reader at 450 nm.^26^

### Statistical analysis

Data was expressed as the mean value ± standard error of mean (SEM). Statistical analysis was performed using GraphPad software (San Diego, CA, United States). Statistical variations between groups were determined using one-way ANOVA analysis (variance for multiple comparisons) followed by a Bonferroni post-test. Values of *p* < 0.05 were considered significant.

## Results

### *In vitro* cell viability

**Figure 1:**
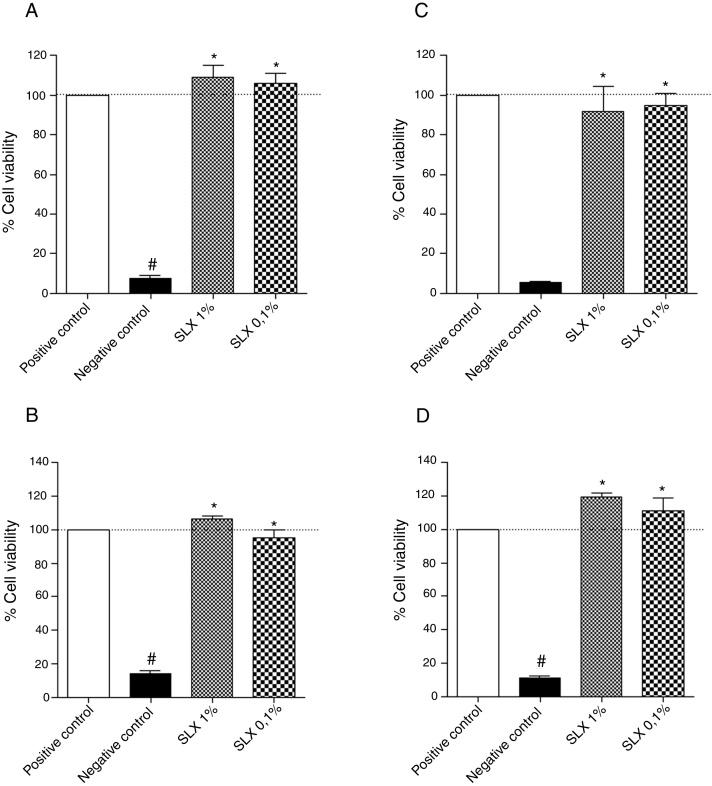
Cell viability. Percentage of viability of NIH-3T3 (undefined) fibroblasts (A–C) and human keratinocytes (B–D) in culture at 24 h (A and B) and 48 h (C and D) relative to the control culture (corresponding to 100% viable cells) by the MTT method, distributed at 1% and 0.1% concentrations of latex serum. Values represent means ± standard error of mean (SEM) of triplicate results. ANOVA statistical test, Bonferroni post-test. *Significant difference (*p* < 0.05) in relation to the negative control. #Significant difference (p < 0.05) between control groups.

Initially, the *in vitro* viability of SLX was evaluated by the MTT colorimetric method using the NIH-3T3 fibroblast cell line and the human keratinocytes. After 24 h of incubation, SLX 1% and 0.1% did not exhibit any cytotoxic effects against fibroblasts compared to basal control (only cell culture medium, considered as 100% viability), showing 109% and 105% viability, respectively (Fig. 1A). Similar results were observed with human keratinocytes exhibiting 106% and 95% viable cells after SLX 1% and 0.1% exposure, respectively (Fig. 1B).

After 48 h of incubation, similar results were observed. Fibroblasts showed viability percentages of 91% and 94% for 1% and 0.1% of SLX, respectively (Fig. 1C), and 119% and 111%, for keratinocytes, respectively (Fig. 1D).

### *In vitro* cell migration/proliferation

**Figure 2:**
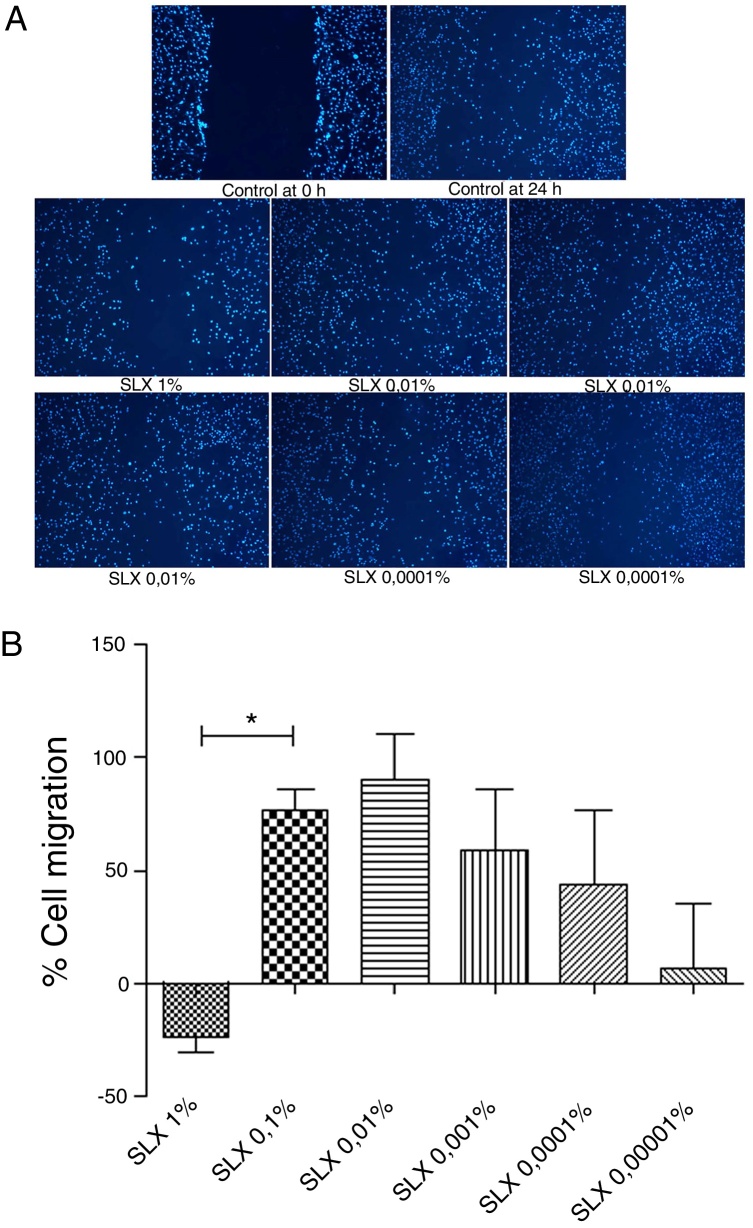
Cell migration. (A) Microscopic fluorescent 4′,6-diamino-2-phenyl-indole (DAPI) image of the scratch assay with human keratinocytes. Cell proliferation/migration in the scratch assay was observed in response to an “artificial lesion” treated with different concentrations of latex serum. (B) Percentage of proliferation/migration of human keratinocytes relative to basal culture medium, set to 0 on the *X*-axis, distributed at various concentrations of latex serum (SLX) treated for 24 h in culture. Values represent mean ± standard error of mean (SEM) of triplicate results. ANOVA statistical test, Bonferroni post-test. *Corresponds to a significant difference (*p* < 0.05) between groups (magnification: 50×).

To evaluate *in vitro* cell migration/proliferation, the scratch assay was performed using human keratinocytes. After 24 h, SLX showed dose dependence increased in human keratinocyte proliferation/migration. The highest stimulatory effect was observed with 0.1% and 0.01% concentrations, and enhanced cell numbers by up to 70%. 1% SLX exhibited an antiproliferative effect (*p* < 0.05) as compared to the number of cells in the control group (Fig. 2A and B).

### *In vivo* evolution of the re-epithelialization process

**Figure 3:**
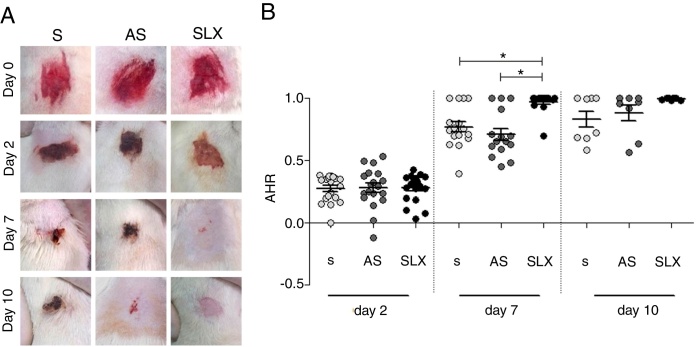
Re-epithelialization. (A) Clinical follow-up of cutaneous abrasions. (B) Quantification of re-epithelialization (by the abrasion healing rate [AHR]) treated with saline (S), antiseptic solution (AS), and latex serum (SLX). ANOVA statistical test, Bonferroni post-test. *Corresponds to a significant difference (*p* < 0.05).

The re-epithelialization process was evaluated by means of a scarring index of abrasions. On the 2nd day of follow-up, all groups that were studied presented similar re-epithelialization (*p* > 0.05). However, on the 7th day, the SLX group presented a higher re-epithelialization rate compared to the S and AS groups (*p* < 0.05). On the 10th day, the abrasions were almost re-epithelized, reaching an average percentage of 83% in the S group, 88% in the AS group, and 99% in the SLX Group (*p* > 0.05) (Fig. 3A and B).

### Histological analysis

**Figure 4:**
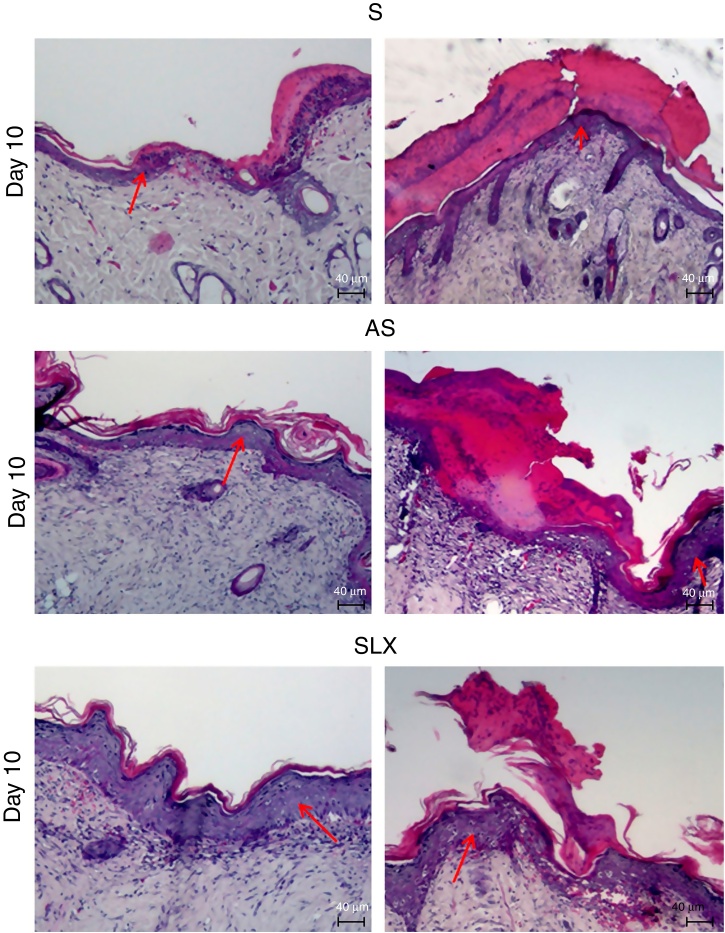
Photomicrography of the exulcerated areas. Photomicrography of the exulcerated areas treated topically with saline (S), antiseptic solution (AS), or latex serum (SLX) on the 10th day of follow-up; samples were stained with hematoxylin–eosin (HE), highlighting the amount of crust and the thickness of the epidermis with a worse and a better photomicrograph in each group and treatment. The red arrows indicate the thickness of the epidermis (magnification: 100×).

Hematoxylin and eosin staining was used for histological analysis. Fig. 4 presents representative images (images with smaller and larger amounts of crust and epidermis were chosen) during different aspects of re-epithelialization of the groups on the 10th day. The SLX group highlighted the smaller amount of crust and a very thick epidermis, with a greater number of layers of keratinocytes in contrast with other groups that still presented a thicker crust and thinner epidermis.

**Figure 5:**
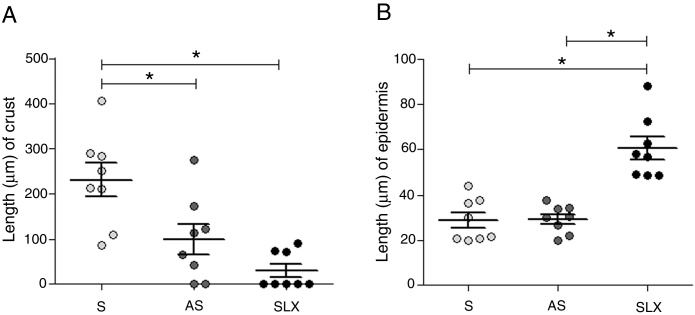
Distribution of the mean thickness (μm) of the crust and epidermis. (A) Distribution of the mean thickness (μm) of the crust and (B) distribution of the mean thickness (μm) of the epidermis of the groups treated with saline (S), antiseptic solution (AS), or latex serum (SLX) on the 10th day of the follow-up. Values represent mean ± standard error of mean (SEM). ANOVA statistical test, Bonferroni post-test. *Corresponds to a significant difference (*p* < 0.05).

Regarding crust thickness, the SLX Group showed a smaller crust thickness than those of the S Group. The AS Group also showed a smaller crust thickness than the S Group (*p* < 0.05; Fig. 5A). The SLX Group had the thickest epidermis among the groups (*p* < 0.05; Fig. 5B).

### Determination of myeloperoxidase

**Figure 6:**
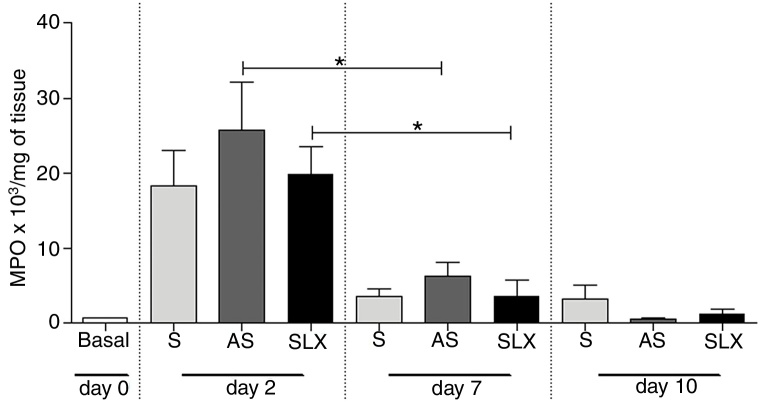
Quantification of the myeloperoxidase enzyme (MPO) of cutaneous abrasions treated topically with saline (S), antiseptic solution (AS), or latex serum (SLX) on the 2nd, 7th, and 10th days of follow-up. Values represent mean ± standard error of mean (SEM). ANOVA statistical test, Bonferroni post-test. *Corresponds to a significant difference (*p* < 0.05).

The evaluation of neutrophil infiltration during the inflammatory process was estimated by myeloperoxidase content in the abrasion biopsies (*n* = 8 per group/day). A high concentration of MPO was observed in all groups on the 2^nd^ day. After seven days post-injury, a decrease in MPO content was observed in all groups, even in SLX and AS (*p* < 0.05). On the 10th day, it showed the same profile as the 7th day. There was no significant difference between the groups in the studied times (*p* > 0.05; Fig. 6).

## Discussion

The primary goal in treating of wounds is to achieve rapid healing with functional and esthetic properties. Superficial wounds like excoriations are frequent in domestic accidents, and home treatments seek rapid pain reduction and wound drying. Also, considering esthetics procedures that include abrasions (such as dermoabrasion, roller, *etc.*) require treatments to reduce the pain and keep the wound moist to avoid unesthetic scars.^27^, ^28^, ^29^

The present study sought to compare the healing efficacy of the product derived from SLX, another product with antiseptic properties used frequently to treat excoriated skin areas, and saline as a natural control. One limitation of this study was the lack of use of a control gel-cream (medium), but there are already a huge number of references about the properties of SLX in wound healing. However, considering the goal of the study was to compare different treatments, the medium type of the commercial antiseptic solution should be considered, which were difficult one to obtain for this study. Considering that there are no studies in the literature about the safety of different concentrations of latex, *in vitro* studies were realized.

According to the WHO/IUIS,^30^ latex contains proteins that are allergenic and are well described in the literature, in total 15 published allergens, 13 of them greater than 10 kDa.^30^, ^31^, ^32^ Thus, the authors of the present work have performed a tangential filtration to process SLX, eliminating proteins up to 10 kDa, leaving the serum nearly free of allergens, but maintaining its properties to heal, as demonstrated by the pre-clinical results. However, for commercial purposes, a notification of the presence of latex should be provided on the label and within the product's instructions for use, for the safety of the patients.

In the cell viability test, SLX 1% and SLX 0.1% presented high viability at 24 h and 48 h (Fig. 1A–D). Mendonça^17^ evaluated the viability of SLX from the *H. brasiliensis* rubber tree at concentrations of 0.1 and 0.01 mg/mL in HEK293T fibroblasts for 48 h and concluded that the latex was not cytotoxic. Andrade^33^ also verified the viability of 3T3-NIH fibroblasts and human keratinocytes for 24 h using SLX protein fraction (F1) from the *H. brasiliensis* rubber tree with a concentration of 0.01%. Corroborating the present findings, these authors concluded that latex protein fraction is viable for use in *in vivo* tissue repair.

Keratinocytes are widely recognized to play an essential role in cutaneous wound healing, and they are considered essential during the proliferative phase because they are responsible for wound closure and re-epithelialization.^34^, ^35^, ^36^, ^37^ After SLX 0.01% was cultured for 24 h, an increase in keratinocyte proliferation/migration was observed in relation to other concentrations (Fig. 2A and B). These results showed an important stimulus of SLX to *in vitro* proliferation/migration.

Having confirmed the ideal doses, safety, and efficacy of SLX in *in vitro* studies, the authors next performed *in vivo* experiments to compare with others products for skin abrasions, AS and S. In the analysis of the re-epithelialization of abrasions, the animals that received SLX treatment on the 7th day presented faster re-epithelialization as compared to the AS and S groups (*p* < 0.05) (Fig. 3B). On the 10th day, practically all of the animals that received SLX treatment were homogeneously re-epithelialized, in contrast with the other groups that had demonstrated a clinical delay in heterogeneous re-epithelialization and a larger crust, although no significant difference was observed (*p* > 0.05) (Fig. 3A). Mendonça^16^ used SLX 0.01% incorporated in carboxy-methylcellulose as a vehicle to treat rabbit ear wounds. Andrade^33^ used SLX 0.01% protein fraction (F1) from *H. brasiliensis* in carboxy-methylcellulose, also to treat skin wounds in diabetic rats. Both studies showed healing properties, suggesting that SLX is able to accelerate wound healing; these findings corroborate with the present results in a cutaneous abrasion model, also with involvement of initial inflammatory signs and accelerated wound healing.

Crust thickness is related to the exudation and inflammatory phases. It was verified that the animals treated with latex were practically without a crust on the 10th day compared with the other groups (Fig. 4). Most of animals treated with SLX presented no crusts from day 5 of follow-up, while animals in the AS and S Groups had thick crusts that could directly imply a delay in re-epithelization. There are no models of excoriation using rats; however, Gupta^38^ used a similar excoriation model, but the lesion was made using a 1.2 cm^2^ sterile scalpel blade during day 0, 1, 2, 4, and 8 of follow-up on BALB/c mice. Untreated animals still had a crust on the eighth day, similar to the present results, except those that underwent SLX treatment.

Moreover, Gupta^38^ observed thinner keratinocyte layers in the untreated group, corroborating the present data, which presented a greater number of keratinocytes layers in SLX Group.

The present authors hypothesize that latex proteins acted more efficiently and quickly to heal wound excoriation.

The inflammatory response is an important step in the healing process because it prepares the injury environment for repair. However, this phase should not be persistent because it may delay re-epithelialization.^39^ Several studies have shown the inflammatory potential of latex from *H. brasiliensis* in the first days after treatment, showing that it is important for debridement *via* the recruitment of inflammatory cells, such as neutrophils and macrophages. The authors also showed that an anti-inflammatory effect after seven to 15 days is important in subsequent stages of healing.^15^, ^40^

Additionally, it was shown that all three groups achieved high levels of MPO in the injury on 2nd day, which is related to the recruitment of neutrophils, but not persistently. On the 7th day, the level of MPO decreased, significantly in the AS and SLX groups (*p* < 0.05) (Fig. 6). However, on 10th day, all groups presented levels of MPO similar to the baseline values.

## Conclusion

Latex serum 1% from *H. brasiliensis* showed favorable results in the healing/re-epithelialization of skin lesions in rats subjected to abrasions, compared with antiseptic (chlorhexidine digluconate 10 mg/mL) and saline solution.

## Financial support

Fundação de Amparo a Pesquisa do Estado de São Paulo (FAPESP-Process 2014/23662-1); Conselho Nacional de Desenvolvimento Científico e Tecnológico (CNPq-Process Master Scholarship 134280/2014-8); Fundação de Apoio ao Ensino Pesquisa e Assistência do Hospital das Clínicas da Faculdade de Medicina de Ribeirão Preto-USP (FAEPA-HCFMRPUSP-Process 108/2014); 10.13039/501100002322Coordenação de Aperfeiçoamento de Pessoal de Nível Superior (CAPES-Process PhD. Scholarship PROEX 7/2017).

## Authors’ contributions

Marcel Nani Leite: Statistical analysis; approval of final version of the manuscript; conception and planning of the study; drafting and editing of the manuscript; collection, analysis, and interpretation of data; participation in design of the study; intellectual participation in the propaedeutic and/or therapeutic conduct of the studied cases; critical review of the literature; critical review of the manuscript.

Saulo Nani Leite: Statistical analysis; approval of final version of the manuscript; conception and planning of the study; drafting and editing of the manuscript; collection, analysis, and interpretation of data; participation in design of the study; intellectual participation in the propaedeutic and/or therapeutic conduct of the studied cases; critical review of the manuscript.

Guilherme Ferreira Caetano: Statistical analysis; approval of final version of the manuscript; conception and planning of the study; drafting and editing of the manuscript; collection, analysis, and interpretation of data; intellectual participation in the propaedeutic and/or therapeutic conduct of the studied cases; critical review of the manuscript.

Thiago Antônio Moretti de Andrade: Statistical analysis; approval of final version of the manuscript; collection, analysis, and interpretation of data; intellectual participation in the propaedeutic and/or therapeutic conduct of the studied cases; critical review of the manuscript.

Márcio Fronza: Statistical analysis; approval of final version of the manuscript; drafting and editing of the manuscript; collection, analysis, and interpretation of data; intellectual participation in the propaedeutic and/or therapeutic conduct of the studied cases; critical review of the manuscript.

Marco Andrey Cipriani Frade: Statistical analysis; approval of final version of the manuscript; conception and planning of the study; drafting and editing of the manuscript; collection, analysis, and interpretation of data; participation in the design of the study; intellectual participation in the propaedeutic and/or therapeutic conduct of the studied cases; critical review of the literature; critical review of the manuscript.

## Conflicts of interest

None declared.
